# Safety and Effectiveness of Irreversible Electroporation in Lymph Node Metastases

**DOI:** 10.1007/s00270-024-03795-w

**Published:** 2024-06-28

**Authors:** Govindarajan Narayanan, Ashwin M. Mahendra, Nicole T. Gentile, Brian J. Schiro, Ripal T. Gandhi, Constantino S. Peña, Madelon Dijkstra

**Affiliations:** 1 Herbert Wertheim College of Medicine, Florida International University, Miami, FL USA; 2 Department of Interventional Oncology, Miami Cancer Institute, Baptist Health South Florida, Miami, FL USA; 3 Department of Interventional Radiology, Miami Cardiac and Vascular Institute, Baptist Health South Florida, Miami, FL USA; 4 Charles E. Schmidt College of Medicine, Florida Atlantic University, Boca Raton, FL USA; 5 Department of Radiology and Nuclear Medicine, Amsterdam UMC, Location VUmc, Cancer Center Amsterdam, Amsterdam, The Netherlands

**Keywords:** Lymph node metastases, Irreversible electroporation (IRE), Tumor response

## Abstract

**Purpose:**

Demonstrating the safety and efficacy of percutaneous irreversible electroporation (IRE) for the treatment of lymph node metastases.

**Materials and Methods:**

An IRB-approved, single-center retrospective review was performed on patients with lymph node metastases gastrointestinal, and genitourinary primary cancers. Primary objective safety was evaluated by assessing complications graded according to the Clavien-Dindo Classification, and efficacy was determined by tumor response on follow-up imaging and local progression-free survival (LPFS). Secondary outcome measures were technical success (complete ablation with an adequate ablative margin > 5 mm), length of hospital stay and distant progression-free survival (DPFS).

**Results:**

Nineteen patients underwent percutaneous IRE between June 2018 and February 2023 for lymph node metastases, close to critical structures, such as vasculature, bowel, or nerves. The technical success was achieved in all cases. Complications occurred in four patients (21.1%), including two self-limiting grade 1 hematomas, a grade 1 abdominal pain, and grade 2 nerve pain treated with medication. Seventeen patients were hospitalized overnight, one patient stayed two nights and another patient stayed fourteen nights. Median follow-up was 25.5 months. Median time to local progression was 24.1 months (95% CI: 0–52.8) with 1-, 2-, and 5-year LPFS of 57.9%, 57.9% and 20.7%, respectively. Median time to distant progression was 4.3 months (95% CI: 0.3–8.3) with 1-, 2-, and 5-year DPFS of 31.6%, 13.2% and 13.2%, respectively.

**Conclusion:**

IRE is a safe and effective minimally-invasive treatment for lymph node metastases in locations, where temperature dependent ablation may be contraindicated. Care should be taken when employing IRE near nerves.

**Graphical Abstract:**

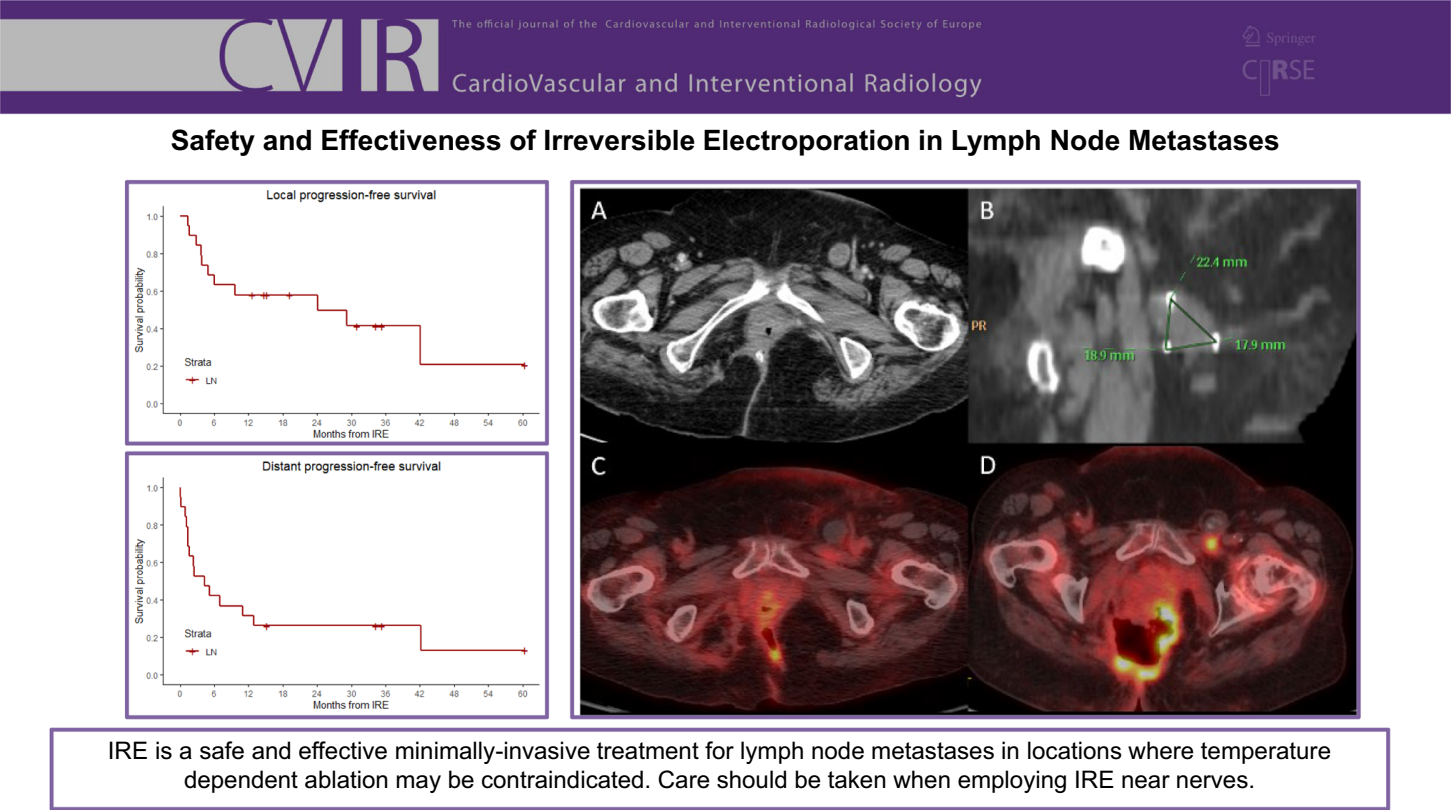

## Introduction

The presence of lymph node metastases is an important prognostic factors for patients with carcinomas [1]. Epithelial cancer metastases typically initiate through lymphatics spreading to draining lymph nodes, with cancer cells from a primary tumor initially settling in a limited number of regional lymph nodes before progressing to other lymph nodes [1, 2]. When the spread of a primary tumor is limited to a single metastatic lymph node, this may identify tumors early in their metastatic potential that may be responsive to local therapy [3]. It has been suggested that limited lymphatic metastasis identifies a subset of metastatic patients amenable to local control, where certain presentations may favor specific local therapies [4–8].

Surgery, radiotherapy and ablative therapies have all been used successfully as local treatment options in the management of lymphatic metastases of various primary cancers [1, 3–5, 8–21]. Many studies have described a strong relationship between the number of nodal metastases and the outcomes of local therapy [8, 9, 11, and 12]. In patients with limited lymph node metastasis, lymphadenectomy has been performed during or following resection of primary urothelial cancer, prostate cancer, and cholangiocarcinoma with improved therapeutic outcomes compared to resection without lymphadenectomy [8–11]. Patients with limited lymph node metastasis secondary to prostate, colorectal, pancreatic, esophageal, and other cancers have demonstrated increased overall survival (OS), progression-free survival (PFS), and local progression-free survival (LPFS) following radiotherapy of metastatic lymph nodes [8, 12, 13]. Studies have shown successful treatment of lymphatic metastasis in high-risk patients using minimally invasive thermal ablative therapies, such as cryoablation, microwave ablation (MWA), and radiofrequency ablation (RFA) [14–22].

While, these thermal ablation modalities cause direct necrosis, irreversible electroporation (IRE) initiates cellular apoptosis by irreversibly damaging the cell membrane with a pulsed, high-voltage, direct current [23, 24]. The use of IRE results in sharper ablation borders and is protective of connective and ductal tissue within its ablation zone, allowing IRE to be used near vasculature and other critical structures sensitive to cryo- or thermal ablation [25, 26]. Current literature on the use of IRE in treating lymph node metastases is limited to an animal study and two case reports. The animal study found IRE to be safe and effective at ablating lymph nodes in porcine models, confirmed by the two case reports describing the successful treatment of lymphatic tumors by IRE [27–29]. The primary objective was to determine safety and efficacy of IRE in the treatment of lymph node metastases.

## Material and Methods

An institutional review board–approved retrospective analysis was performed at a single facility on patients with lymph node metastases of gastrointestinal, and genitourinary primary cancers.

### Patients Selection and Data Collection

Patients who underwent IRE for lymph node metastases with at least one follow-up imaging exam after ablation were included. Lymph node metastases were defined as the presence of FDG avid lymph nodes on PET-CT prior to ablation. Patients with still active and FDG avid lymph node metastases after other standard treatments such as chemotherapy and radiation therapy or ineligible for other local treatment options were considered as potential candidates for IRE. IRE was performed due to the proximity of the nodal metastases to critical structures or vasculature. Patients had sufficient kidney, liver, and bone marrow function, and were medically fit to undergo general anesthesia. Patient and tumor characteristics were gathered from medical records and evaluated at baseline.

### Irreversible Electroporation Procedure

The procedures were performed percutaneously under general anesthesia with complete muscle relaxation. A preprocedure contrast-enhanced CT scan was obtained to ensure suitable patient positioning and to facilitate access to the target lesion. The NanoKnife™ device (AngioDynamics, Queensbury, New York) was used for this study. The IRE was set up to produce 70-microsecond high-voltage (1,500–3,000 V) direct current (25–45A) electrical pulses. Typically, 70 pulses were delivered in seven sets of 10 pulses between paired unipolar electrodes. The voltage setting for each electroporation was determined by the distance between each pair of electrodes, with the intent to generate at least 1,000 V to a maximum of 1,500 V per cm between the electrodes. No tissue separation maneuvers were used to protect structures adjacent to the IRE electrodes. CT guidance was used to advance the electrodes percutaneously, and current was applied when CT had confirmed adequate position. The generator was programmed to stop delivery and recharge if the current flow exceeded 48 Amps.

At the time of ablation completion, a contrast-enhanced CT scan was performed to confirm that the entire target had been ablated, to assess blood vessels proximal to the treatment zone, and evaluate for any urgent post-procedural complications. Patients were then awakened and transferred to the recovery area for at least 4 h. The following day, patients were examined and routine laboratory tests, including complete blood count and complete serum chemistry profile, were evaluated. Patients were discharged when stable and showing minimum risk for postprocedural infections and/or complications. Follow-up scans were obtained at one, three, six and 12 months post procedure and thereafter as clinically indicated. Repeat IRE treatment was offered if follow-up imaging showed partial response, stable or progressive disease according to mRECIST criteria [30].

### Outcome Measures

Safety was evaluated by assessing adverse effects recorded following the procedure. All adverse effects potentially related to the procedure were graded according to the Clavien-Dindo Classification [31]. Tumor response was determined by comparing enhancement and/or FDG uptake between the latest pre- and earliest post- ablation contrast enhanced CT or MRI with diffusion-weighted sequences obtained after a at least one-month post ablation, and after at least three months with PET-CT. A complete response was defined as a lack of enhancement or FDG uptake at the tumor location on earliest post ablation imaging. A partial response was defined as residual enhancement or FDG uptake at the tumor location. Stable disease was defined as no change in enhancement or FDG uptake at the tumor location on post ablation imaging. Progressive disease was defined as an increase in enhancement or FDG uptake at the tumor location on post ablation imaging. Efficacy was determined by uptake or lack of uptake on PET-CT and/or enhancement on CT/MRI, and LPFS of the treated lymph node was recorded. Secondary outcome measures were technical success, length of hospital stay and distant progression-free survival (DPFS) Time-to-event endpoints were all analyzed using Kaplan Meier curves [30, 32]. Technical success was defined as complete ablation with an adequate ablative margin (intentional tumor free ablation margin > 5mm). Data analysis was performed using Excel (Microsoft), SPSS® Version 28.0 (IBM®, Armonk, New York, USA) and R version 4.2.1. (R Foundation, Vienna, Austria), and results were tabulated [33, 34].

## Results

### Patient and Tumor Characteristics

Nineteen patients [nine males and ten females] with 24 lymph node metastases underwent percutaneous IRE between June 2018 and February 2023. Patient oncologic information and tumor characteristics are shown in Table 1. The median patient age was 64.4 years (range 33–87). Patients had tumors of primary cholangio-/gallbladder carcinoma (*n* = 3), gastric cancer (*n* = 1), duodenal/jejunum carcinoma (*n* = 2), colorectal cancer (*n* = 7), urothelial cancer (*n* = 3) and prostate cancer (*n* = 3). Sites of lymphatic metastasis varied between patients, but all sites were close to critical structures sensitive to cryo- or thermal ablation such as vasculature (*n* = 9), bowel (*n* = 9), and nerves (*n* = 1). A total of 18 out of 19 patients had undergone surgical resection of the primary tumor prior to ablation (94.7%), 15 patients had completed at least one cycle of systemic therapy (78.9%), 5 patients received radiotherapy (26.3%) and 2 patient thermal ablation for distant metastases (10.5%). The average tumor size was 21.3 mm (range 6–35).

**Table 1:** Patient and tumor characteristics

Patient characteristics (*N* = 19)	Value (*N*, %)
Age in years (mean, range)	64.4 (33–87)
Sex	
Male	9
Female	10
Primary cancer site	
Bile duct/gallbladder	3 (15.8%)
Gastric	1 (5.3%)
Small intestine	2 (10.5%)
CRC	7 (36.8%)
Urothelial	3 (15.8%)
Prostate	3 (15.8%)
TNM staging *	
Primary tumor	
T1	3 (15.8%)
T2	4 (21.1%)
T3	7 (36.8%)
T4	4 (21.1%)
Regional lymph nodes	
N0	8 (42.1)
N1	7 (36.8%)
N2	2 (10.5%)
NX	1 (5.3%)
Distant metastases	
M0	14 (73.7)
M1	3 (15.8%)
MX	1 (5.3%)
Previous treatment (including primary)	
Surgery	18 (94.7%)
Chemotherapy	15 (78.9%)
Radiotherapy	5 (26.3%)
Thermal ablation	2 (10.5%)
Number of lymph nodes	
1	15 (78.9%)
2	3 (15.8%)
3	1 (5.3%)
Tumor characteristics (*N* = 24)	
Location lymph node	
Abdominal	17 (70.8%)
Pelvic	4 (16.7%)
Inguinal	3 (12.5%)
Mean tumor diameter in mm ( range)	21.3 (6–35)

*CRC* colorectal cancer

^*^1 missing (5.3%)

Median length of patient follow-up was 25.5 (range 4.1–60.4) months. Following IRE, twelve patients were treated with systemic therapy and eight patients received other locoregional therapies for distant metastases.

### Adverse Events

Four patients experienced complications (21.1%). One patient had Clavien-Dindo Grade 2, post-operative, ipsilateral leg pain. This patient had an IRE of a pelvic sidewall lymph node abutting the sacral nerve (0 mm). Upon recovery from anesthesia after the procedure, the patient reported severe right leg pain without any motor deficits. An MRI of the lumbar spine was negative for any acute pathology. Pain management was consulted and the patient’s pain was treated with diazepam, oxycodone, gabapentin, hydromorphone, and amitriptyline. Complaints improved along the course of the 14-night stay. At discharge the patient continued their medication regimen. Currently, the patient ambulates with the assistance of a walker due to pain. Two other patients demonstrated with asymptomatic, Clavien-Dindo Grade 1, trace hematomas on post ablation imaging, which spontaneously resolved by the next follow-up imaging study. One patient with a retrocrural lymph node presented with a grade 1 abdominal pain, chest X-ray showed no signs of pneumothorax and with pain medication the patient recovered. Length of hospital stay was fourteen nights for the patient with nerve pain (5.3%), and two nights in the patient with abdominal pain (5.3%), for all other patients the length of hospital stay was one night (89.5%).

### Tumor Response

**Fig. 1:**
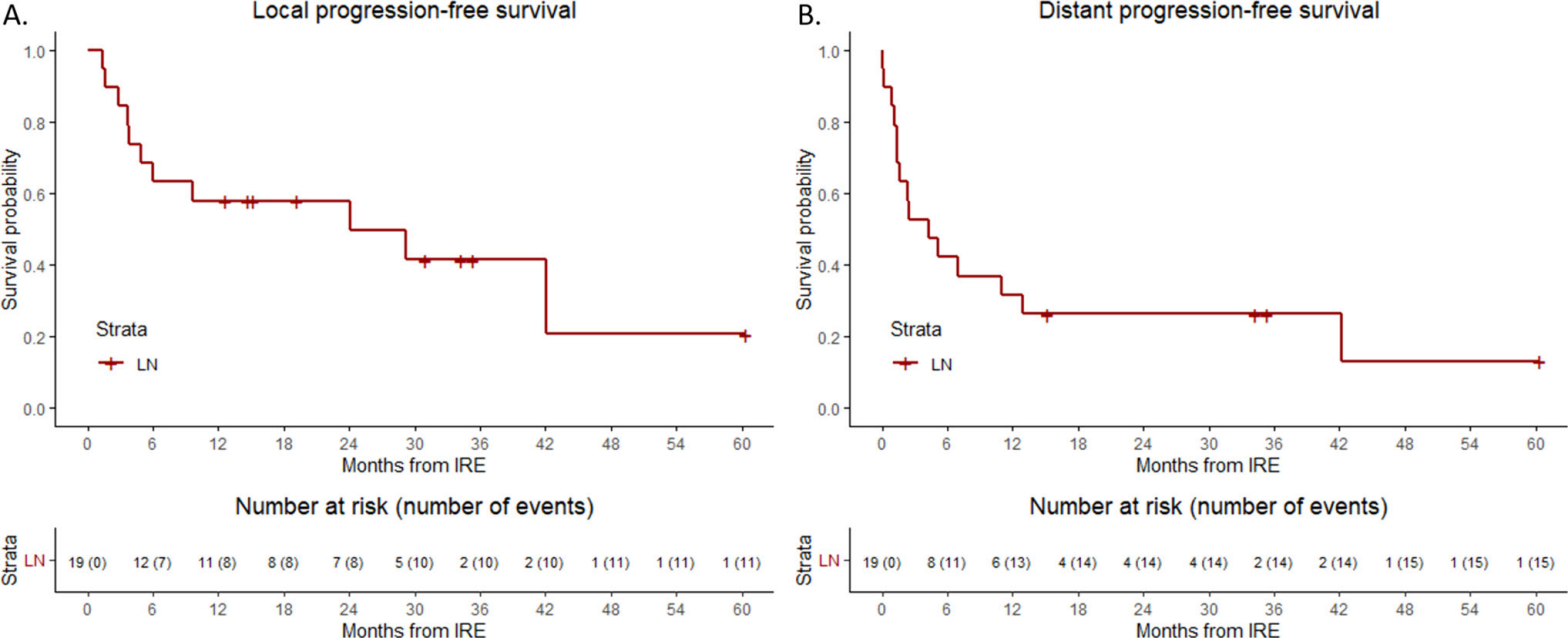
Kaplan–Meier survival curves of (**A**) local progression-free survival (LPFS) and (**B**) distant progression-free survival after irreversible electroporation (IRE) per patient

**Fig. 2:**
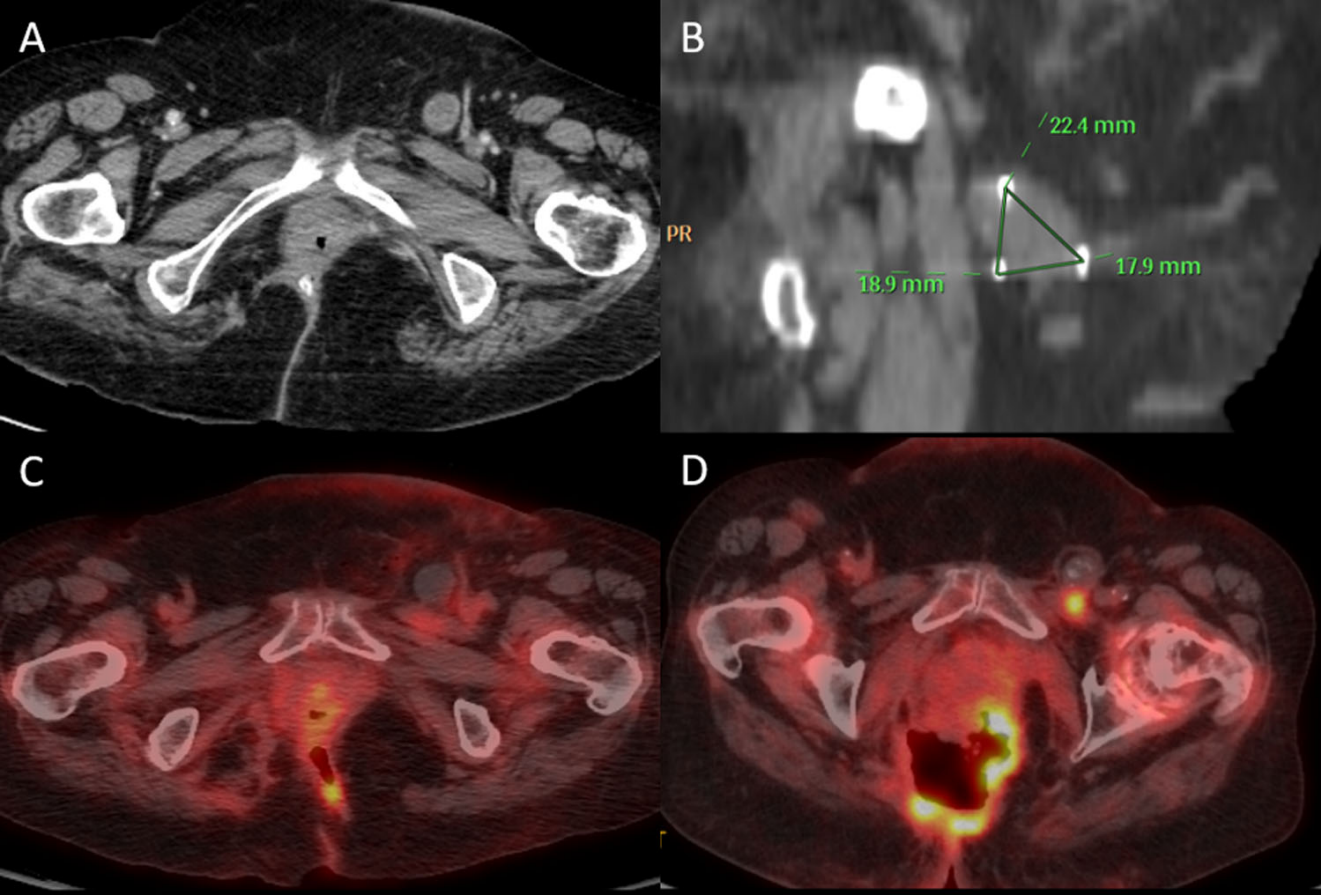
A 66-year-old female admitted with rectal pain. Past medical history of adenocarcinoma of the rectum diagnosed in 2019, underwent loop sigmoid colostomy, completed radiation and is receiving adjuvant chemotherapy (FOLFOX) upon treatment. (**A**) Abdominopelvic CT shows a left inguinal lymph node measuring up to 2.7 cm concerning for metastasis. Ultrasound-guided biopsy of the left inguinal lymph node with frozen samples was positive for adenocarcinoma. (**B**) Intraprocedural CT demonstrating intermittent placement of three 17-gauge NanoKnife IRE ablation needles. (**C**) Follow-up PET-CT the following day showing lack of FDG-uptake of the treated lymph node. (**D**) Follow-up PET-CT after 29 months showing FDG-uptake of the treated lymph node

Technical success was obtained in 100% of cases. Twenty of the 24 lymph nodes showed a reduction in tumor size (83.3%) at first follow-up conducted after at least 1-month including complete and partial responses, one lymph node showed stable disease and two lymph nodes showed progression in tumor sizes. Eleven of the nineteen patients (57.9%) experienced local recurrence of disease. Median time to local progression of disease was 24.1 months (95% CI: 0–52.8) with 1-, 2-, and 5-year LPFS of 57.9%, 57.9% and 20.7%, respectively (Fig. 1A**)**. One patient received secondary IRE treatment for residual disease which showed progression on follow up imaging 8.5 months after initial IRE. This patient received repeat IRE for a tumor that demonstrated a partial response to the original treatment site and has since maintained a complete response with no evidence of local recurrence over 2 years. Two other patients received two additional IRE treatments for local recurrent lymph node metastases. Median distant progression was 4.3 months (95% CI: 0.3–8.3) with 1-, 2-, and 5-year DPFS of 31.6%, 13.2% and 13.2%, respectively (Fig. 1B**)**. An example case is shown in Fig. 2.

## Discussion

Metastases are one of the most important prognostic factors for decreased overall survival (OS) in cancer patients, with lymphatic vessels as the most common conduit for tumor metastasis [1]. Local methods of obtaining tumor control, including surgery, radiotherapy, and ablative therapies, have all been used to manage lymphatic metastasis [1, 4, 5, 8–21]. Technical success was achieved in all patients, 84.2% of patients initially showed a reduction in tumor size. The 1-, 2-, and 5-year LPFS rates were estimated at 57.9%, 57.9% and 20.7%, respectively. Complications occurred in four patients, including two self-limiting grade 1 hematomas, a grade 1 abdominal pain, and grade 2 nerve pain treated with medication.

The cohort in this study constitutes a subset of patients for whom alternative local treatment modalities are not viable due to the close proximity to critical anatomical structures. Despite, the relatively elevated occurrence of local recurrences, the median time to local progression was 24.1 months, which presents IRE as a viable treatment option. IRE is technically feasible and efficacious for lymph node metastases, with reduced disease progression compared to palliative treatment [35]. IRE is repeatable for cases of recurrent disease, as exemplified in three patients. Considering that the lymph node metastases were in proximity to vital structures like vasculature, bowel, and nerves, with just one grade 2 nerve injury, IRE has a highly safety profile.

Research describing lymph node metastases treatment is sparse, yet local therapy has shown improved oncologic outcomes in selected cohorts [8, 9, 11, and 12]. Five-year OS among colon and esophageal cancer patients with lymph node metastases was found to be 74.6% and 35%, respectively [6, 7]. Jingu et al. conducted a retrospective review of 35 patients with primary esophageal cancer who received radio-chemotherapy for recurrent lymph node metastases following esophagectomy with lymph node dissection [5]. The study reports rates of 5-year irradiated-field control, OS, and PFS of 59.9%, 39.2%, and 31.0%, respectively. Patients experienced a substantially improved three-year OS of 50.3%, compared to 3-year OS rates of 4.3–35.7% reported in prior studies investigating radiotherapy with and without concurrent chemotherapy for post-operative, recurrent esophageal cancer. A recent study reported a significantly prolonged OS in patients with oligo non-regional lymph node metastases following surgical resection, thermal ablation or radiotherapy compared to palliative treatment alone in selected cases (73 vs. 23 months) [35–37].

Percutaneous ablation offers a minimally invasive approach that is more easily repeated over prior treatment sites [15–17, 22]. Recent studies have expanded the role of thermal ablation in the treatment of lymphatic metastases. A retrospective study of eight patients who underwent RFA for 20 cervical lymph node metastases of thyroid carcinoma reported no complications and no evidence of local recurrence with mean follow-up of nine months [16]. A study of MWA in 11 patients with 24 cervical metastases of primary papillary thyroid carcinoma evaluated outcomes over a 32-month mean follow-up period [17]. No patients demonstrated recurrent lesions or evidence of minor or major complications.

Cryoablation has been more extensively researched in the treatment of lymphatic metastases. Cryoablation allows for easier visualization of the ablation zone on CT, ensuring coverage of the tumor [18–20]. The first study published on this topic enrolled 18 patients, ablated 27 lymph nodes and reported a mean follow-up of 15 months. Preliminary outcomes showed only one lymph node with progressive disease and two minor complications of obturator nerve paresis [18]. Two larger studies demonstrated that cryoablation of lymph nodes can be performed with excellent LPFS [19, 20]. A single-center retrospective study of cryoablation performed on 56 meta-synchronous lymphatic metastases in 29 patients reported a three-year LPFS of 94.3% [20]. There were two incidents of local tumor progression during the follow-up period (median 23 months) and two incidents of transient nerve palsies. The second study reported local progression in 12 of the 65 treated lymph nodes during a similar follow-up period (median 25 months) [19]. Two major complications of bleeding and pneumothorax occurred and a LPFS rate of 82% was reported at 11 months in the 55 patient sample. The reduced survival and increased incidence of major complications in this study over the former may be explained in part by the deeper location of treated lymph nodes [19, 20]. A bi-institutional retrospective review comparing cryoablation to RFA in metastatic lymph nodes concluded that the two techniques are equally effective and safe [21]. Three out of 26 tumors treated by RFA and one out of sixteen tumors treated by cryoablation showed signs of local progression. The one-year LPFS following RFA and cryoablation were 93.8% and 88.5%, respectively. No complications were reported.

Unlike these thermal ablation modalities, IRE initiates cellular apoptosis by irreversibly damaging the cell membrane with a pulsed, high-voltage, direct current [23, 24]. IRE results in sharper ablation borders and is protective of connective and ductal tissue within its ablation zone, allowing IRE to be used near vasculature and other critical structures sensitive to cryo- or thermal ablation [25, 26]. This may lend IRE an advantage in the ablation of lymph nodes as they tend to reside near large vessels. In all studies of cryoablation, RFA, and MWA discussed, methods of thermal-insulation displacement maneuvers were used to protect critical structures. In select patients, IRE can circumvent the need for such maneuvers.

Limitations of this study include the single-center retrospective nature. In addition, there is no control group for comparison. The heterogeneity of primary cancers and variety in tumor locations complicate generalizing oncological outcomes to specific patients. The relative high number of local recurrences were indicated as new spread of the primary tumor, not a lack of efficacy of IRE, considering the high number of complete responses and extensive median time to local tumor progression. However, with early progression of disease, shown with the low median distant progression, might argue the additive value of IRE for metastatic lymph nodes.

## Conclusion

In certain cases, percutaneous IRE might be useful as an adjunctive or alternative modality to surgery, radiation, or other methods of ablation in controlling lymphatic metastases, especially when tumors are adjacent to thermally sensitive critical structures. The findings show that IRE is a technically feasible, safe, and effective treatment, and additional care should be taken when ablating lymph nodes near nerves with IRE.
